# Relations in biomedical ontologies

**DOI:** 10.1186/gb-2005-6-5-r46

**Published:** 2005-04-28

**Authors:** Barry Smith, Werner Ceusters, Bert Klagges, Jacob Köhler, Anand Kumar, Jane Lomax, Chris Mungall, Fabian Neuhaus, Alan L Rector, Cornelius Rosse

**Affiliations:** 1 Institute for Formal Ontology and Medical Information Science, Saarland University, D-66041 Saarbrücken, Germany; 2 Department of Philosophy, University at Buffalo, Buffalo, NY 14260, USA; 3 European Centre for Ontological Research, Saarland University, D-66041 Saarbrücken, Germany; 4 Department of Genetics, University of Leipzig, D-04103 Leipzig, Germany; 5 Rothamsted Research, Harpenden, AL5 2JQ, UK; 6 European Bioinformatics Institute, Hinxton, CB10 1SD, UK; 7 HHMI, Department of Molecular and Cellular Biology, University of California, Berkeley, CA 94729, USA; 8 Department of Computer Science, University of Manchester, M13 9PL, UK; 9 Department of Biological Structure, University of Washington, Seattle, WA 98195, USA

## Abstract

To enhance the treatment of relations in biomedical ontologies we advance a methodology for providing consistent and unambiguous formal definitions of the relational expressions used in such ontologies in a way designed to assist developers and users in avoiding errors in coding and annotation. The resulting Relation Ontology can promote interoperability of ontologies and support new types of automated reasoning about the spatial and temporal dimensions of biological and medical phenomena.

## Abstract 2

To enhance the treatment of relations in biomedical ontologies we advance a methodology for providing consistent and unambiguous formal definitions of the relational expressions used in such ontologies in a way designed to assist developers and users in avoiding errors in coding and annotation.

## Background

### Controlled vocabularies in bioinformatics

The background to this paper is the now widespread recognition that many existing biological and medical ontologies (or 'controlled vocabularies') can be improved by adopting tools and methods that bring a greater degree of logical and ontological rigor. We describe one endeavor along these lines, which is part of the current reform efforts of the Open Biomedical Ontologies (OBO) consortium [1,2] and which has implications for ontology construction in the life sciences generally.

The OBO ontology library [1] is a repository of controlled vocabularies developed for shared use across different biological and medical domains. Thus the Gene Ontology (GO) [3,4] consists of three controlled vocabularies (for cellular components, molecular functions, and biological processes) designed to be used in annotations of genes or gene products. Some ontologies in the library - for example the Cell and Sequence Ontologies, as well as the GO itself - contain terms which can be used in annotations applying to all organisms. Others, especially OBO's range of anatomy ontologies, contain terms applying to specific taxonomic groups such as fly, fungus, yeast, or zebrafish.

Controlled vocabularies can be conceived as graph-theoretical structures consisting on the one hand of *terms *(which form the nodes of each corresponding graph) linked together by means of edges called *relations*. The ontologies in the OBO library are organized in this way by means of different types of relations. OBO's Mouse Anatomy ontology, for example, uses just one type of edge, labeled *part_of*. The GO currently uses two, labeled *is_a *and *part_of*. The *Drosophila *Anatomy ontology includes also a *develops_from *link. Other OBO ontologies include further links, for example (in the Sequence Ontology) *position_of *and *disjoint_from*. The National Cancer Institute (NCI) Thesaurus adds many additional links, including *has_location *for anatomical structures and different *part_of *relations for structures and for processes.

The problem is that when OBO and similar ontologies incorporate such relations they typically do so in informal ways, often providing no definitions at all, so that the logical interconnections between the various relations employed are unclear, and even the relations *is_a *and *part_of *are not always used in consistent fashion both within and between ontologies. Our task in what follows is to rectify these defects, drawing on the requirements analysis presented in [5].

Of the criteria that ontologies must currently satisfy if they are to be included in the OBO library, the most important for our purposes are: first, inclusion of textual definitions or descriptions designed to ensure that the precise meanings of terms as used within particular ontologies will be clear to a human reader; second, employment of a standard syntax, such as the OWL or OBO flatfile syntax; third, orthogonality to the other ontologies already included in the library. These criteria are designed to support the integration of OBO ontologies, above all by ensuring the compatibility of ontologies pertaining to an identical subject matter. OBO has now added a fourth criterion to assist in achieving such compatibility, namely that the relations (edges) used to connect terms in OBO ontologies should be applied in ways consistent with their definitions as set forth in this paper.

The Relation Ontology offered here is designed to put flesh on this criterion. How, exactly, should *part_of *or *located_in *be defined in order to ensure maximally reliable curation of each single ontology while at the same time guaranteeing maximal leverage in building a solid base for life-science knowledge integration in general? We describe a rigorous methodology for providing an answer to this question and illustrate its use in the construction of an easily extendible list of ten relations of a type familiar to those working in the bio-ontological field. This list forms the core of the new OBO Relation Ontology. What is distinctive about our methodology is that, while the relations are each provided with rigorous formal definitions, these definitions can at the same time be formulated in such a way that the underlying technical details remain invisible to ontology authors and curators.

### Shortcomings of biomedical ontologies

While considerable effort has been invested in the formulation and definition of terms in biomedical ontologies, too little attention has been paid in the ontological literature to the associated relations. A number of characteristic types of shortcomings of controlled vocabularies can be traced back especially to the neglect of issues of formal structure in the treatment of relations [5-10]. To take just one example, the pre-2004 versions of GO allowed at least three different readings of the expression 'part of' as representing simultaneously: inclusion relations between vocabularies; a relation of possible parthood between biological entities; a relation of necessary parthood between biological entities. As was shown in [6], this coexistence of conflicting readings meant that three of the four rules given in the then effective documentation for reasoning with GO's hierarchies were logically incorrect.

Another characteristic family of problems turns on the paucity of resources for expressing relations in ontologies like GO. For example, because GO has no direct means of asserting location relations, it must capture such relations indirectly by constructing new terms involving syntactic operators such as 'site of', 'within', 'extrinsic to', 'space', 'region', and so on. It then simulates assertions of location by means of '*is_a*' and '*part_of*' statements involving such composites, for example in:

extracellular region is_a cellular component

extrinsic to membrane part_of membrane

both of which are erroneous. Additional problems arise from the fact that GO's *extracellular region *and *extracellular space *are both specified in their definitions as referring to the space (how large a space?) external to the outermost structure of a cell.

Another type of problem turns on the failure to distinguish relational expressions which, though closely related in meaning, are revealed to be crucially distinct when explicated in the formally precise way that is demanded by computer implementations. An example is provided by the simultaneous use in OBO's Cell Ontology of both *derives_from *and *develops_from *while no clear distinction is drawn between the two [11]. This problem is resolved in the treatment of derivation and transformation below, and has been correspondingly corrected in versions 1.14 and later of the Cell Ontology.

Efforts to improve GO from the standpoint of increased formal rigor have thus far been concentrated on re-expressing the existing GO schema in a description logic (DL) framework. This has allowed the use of a DL-reasoner that can identify certain kinds of errors and omissions, which have been corrected in later versions of GO [12]. DLs, however, can do no more than guarantee consistent reasoning according to the definitions provided to them. If the latter are themselves problematic, then a DL can do very little to identify or resolve the problems which result. Here, accordingly, we take a more radical approach, which consists in re-examining the basic definitions of the relations used in GO and in related ontologies in an attempt to arrive at a methodology which will lead to the construction of ontologies which are more fundamentally sound and thus more secure against errors and more amenable to the use of powerful reasoning tools. This approach is designed also to be maximally helpful to biologists by avoiding the problems which arise by virtue of the fact that the syntax favored in the DL-community is of a type which can normally be understood only by DL-specialists.

### A theory of classes and instances

The relations in biological ontologies connect classes as their relata. The term 'class' here is used to refer to what is general in reality, or in other words to what, in the knowledge-representation literature, is typically (and often somewhat confusingly [13]) referred to under the heading 'concept' and in the literature of philosophical ontology under the headings 'universal', 'type' or 'kind'. Biological classes are in first approximation those classes which have been implicitly sanctioned through usage of the corresponding general terms in the biological literature, for example *cell *or *fat body development*.

Our task is to develop a suite of coherently defined bio-ontological relations that is sufficiently compact to be easily learned and applied, yet sufficiently broad in scope to capture a wide range of the relations currently coded in standard biomedical ontologies. Unfortunately the realization of this task is not a trivial matter. This is because, while the terms in biomedical ontologies refer exclusively to classes - to what is *general *in reality - we cannot define what it means for one class to stand to another, for example in the *part_of *relation, without taking the corresponding instances into account [6]. Here the term 'instance' refers to what is *particular *in reality, to what are otherwise called 'tokens' or 'individuals' - entities (including processes) which exist in space and time and stand to each other in a variety of instance-level relations. Thus we cannot make sense of what it means to say *cell nucleus part_of cell *unless we realize that this is a statement to the effect that each instance of the class *cell nucleus *stands in an instance-level part relation to some corresponding instance of the class *cell*.

This dependence of class-relations on relations among corresponding instances has long been recognized by logicians, including those working in the field of description logics, where the (*all - some*) form of definition we utilize below has been basic to the formalism from the start [14]. Definitions of this type were incorporated also into the DL-based GALEN medical ontology [15], though the significance of such definitions, and more generally of the role of instances in defining class relations, has still not been appreciated in many user communities.

It is also characteristically not realized that talk of classes involves in every case a more-or-less explicit reference to corresponding instances. When we assert that one class stands in an *is_a *relation to another (that is, that the first is a subtype of the second), for example, that *glucose metabolism is_a carbohydrate metabolism*, then we are stating that instances of the first class are *ipso facto *instances of the second. When we are dealing exclusively with *is_a *relations there is little reason to take explicit notice of this two-sided nature of ontological relations. When, however, we move to ontological relations of other types, then it becomes indispensable, if many characteristic families of errors are to be avoided, that the implicit reference to instances be taken carefully into account.

### Types of relations

We focus here exclusively on genuinely ontological relations, which we take to mean relations that obtain between entities in reality, independently of our ways of gaining knowledge about such entities (and thus of our experimental methods) and independently of our ways of representing or processing such knowledge in computers. A relation like *annotates *is not ontological in this sense, as it links classes not to other classes in nature but rather to terms in a vocabulary that we ourselves have constructed. We focus also on general-purpose relations - relations which can be employed, in principle, in all biological ontologies - rather than on those specific relations (such as *genome_of *or *sequence_of *employed by OBO's Sequence Ontology) which apply only to biological entities of certain kinds. The latter will, however, need to be defined in due course in accordance with the methodology advanced here.

The ontologies in OBO are designed to serve as controlled vocabularies for expressing the results of biological science. Sentences of the form '*A relation B*' (where '*A*' and '*B*' are terms in a biological ontology and '*relation*' stands in for '*part_of*' or some similar expression) can thus be conceived as expressing general statements about the corresponding biological classes or types. Assertions about corresponding instances or tokens (for example about the mass of this particular specimen in this particular Petri dish), while indispensable to biological research, do not belong to the general statements of biological science and thus they fall outside the scope of OBO and similar ontologies as these are presented to the user as finished products.

Yet such assertions are still relevant to ontologies. For it turns out that it is only by means of a detour through instances that the definitions and rules for coding relations between classes can be formulated in an intuitive and unambiguous - and thus reliably applicable - way.

We can distinguish, in fact, the following three kinds of binary relations:

<class, class>: for example, the *is_a *relation obtaining between the class *SWR1 complex *and the class *chromatin remodeling complex*, or between the class *exocytosis *and the class *secretion*;

<instance, class>: for example, the relation **instance_of **obtaining between this particular vesicle membrane and the class *vesicle membrane*, or between this particular instance of mitosis and the class *mitosis*;

<instance, instance>: for example, the relation of instance-level parthood (called **part_of **in what follows), obtaining between this particular vesicle membrane and the endomembrane system in the corresponding cell, or between this particular M phase of some mitotic cell cycle and the entire cell cycle of the particular cell involved.

Here classes and the relations between them are represented in *italic*; all other relations are picked out in **bold**.

### Continuants and processes

The terms 'continuant' and 'process' are generalizations of GO's 'cellular component' and 'biological process' but applied to entities at all levels of granularity, from molecule to whole organism. Continuants are those entities which endure, or continue to exist, through time while undergoing different sorts of changes, including changes of place. Processes are entities that unfold themselves in successive temporal phases [16]. The terms 'continuant' and 'process' thus correspond to what, in the literature of philosophical ontology, are known respectively as 'things' (objects, endurants) and 'occurrents' (activities, events, perdurants) respectively. A continuant is what changes; a process is the change itself. The continuant classes relevant to biological ontologies include *molecule*, *cell*, *membrane*, *organ*; the process classes include *ion transport*, *cell division*, *fat body development*, *breathing*.

To formulate precise definitions of the <class, class> relations which form the target of ontology construction in biology we will need to employ a vocabulary that allows reference both to classes and to instances. For this we take advantage of the machinery of logic, and more specifically of the standard device of variables and quantifiers [17], using different sorts of variables to range across the classes and instances of continuants and processes, spatial regions and temporal instants, respectively. For the sake of intelligibility we use a semi-formal syntax, which can, however, be translated in a simple way into standard logical notation.

We use variables of the following sorts:

*C*, *C*_1_, ... to range over continuant classes;

*P*, *P*_1_, ... to range over process classes;

*c*, *c*_1_, ... to range over continuant instances;

*p*, *p*_1_, ... to range over process instances;

*r*, *r*_1_, ... to range over three-dimensional spatial regions;

*t*, *t*_1_, ... to range over instants of time.

In an expanded version of our formal machinery we will need also to incorporate further variables, ranging for example over temporal intervals, biological functions, attributes and values.

Note that continuants and processes form non-overlapping categories. This means in particular that no subtype or parthood relations cross the continuant-process divide. The tripartite structure of the GO recognizes this categorical exclusivity and extends it to functions also.

Continuants can be *material *(a mitochondrion, a cell, a membrane), or *immaterial *(a cavity, a conduit, an orifice), and this, too, is an exclusive divide. Immaterial continuants have much in common with spatial regions [18]. They are distinguished therefrom, however, in that they are *parts of organisms*, which means that, like material continuants, they move from one spatial region to another with the movements of their hosts.

The three-dimensional continuants that are our primary focus here typically have a top and a bottom, an anterior and a posterior, an interior and an exterior. Processes, in contrast, have a beginning, a middle and an end. Processes, but not continuants, can thus be partitioned along the time axis, so that, for example, your youth and your adulthood are temporal parts of that biological process which is your life.

As child and adult are continuants, so youth and adulthood are processes. We are thus clearly dealing here with two complementary - space-focused and time-focused - views of the same underlying subject matter, with determinate logical and ontological connections between them [16]. The framework advanced below allows us to capture these connections by incorporating reference to spatial regions and to temporal instants, both of which can be thought of as special kinds of instances.

We shall also need to distinguish two kinds of instance-level relations: those (applying to continuants) whose representations must involve a temporal index, and those (applying to processes) which do not. Note that the drawing of this distinction is still perfectly consistent with the fact that processes themselves occur in time, and that processes may be built out of successive subprocesses instantiating distinct classes.

### Primitive instance-level relations

We cannot, on pain of infinite regress, define all relations, and this means that some relations must be accepted as primitive. The relations selected for this purpose should be self-explanatory and they should as far as possible be domain-neutral, which means that they should apply to entities in all regions of being and not just to those in the domain of biology.

Our choice of primitive relations is as follows:

*c ***instance_of ***C ***at ***t *- a primitive relation between a continuant instance and a class which it instantiates at a specific time

*p ***instance_of ***P *- a primitive relation between a process instance and a class which it instantiates holding independently of time

*c ***part_of ***c*_1 _**at ***t *- a primitive relation between two continuant instances and a time at which the one is part of the other

*p ***part_of ***p*_1_, *r ***part_of ***r*_1 _- a primitive relation of parthood, holding independently of time, either between process instances (one a subprocess of the other), or between spatial regions (one a subregion of the other)

*c ***located_in ***r ***at ***t *- a primitive relation between a continuant instance, a spatial region which it occupies, and a time

*r ***adjacent_to ***r*_1 _- a primitive relation of proximity between two disjoint continuants

*t ***earlier ***t*_1 _- a primitive relation between two times

*c ***derives_from ***c*_1 _- a primitive relation involving two distinct material continuants *c *and *c*_1_

*p ***has_participant ***c ***at ***t *- a primitive relation between a process, a continuant, and a time

*p ***has_agent ***c ***at ***t *- a primitive relation between a process, a continuant and a time at which the continuant is causally active in the process

This list includes only those <instance-instance> relations, together with one <instance-class> relation, which are needed for defining the <class, class> relations which are our principal target in this paper. The items on the list have been selected because they enjoy a high degree of intelligibility to the human authors and curators of biological ontologies. For purposes of supporting computer applications, however, the meanings of the corresponding relational expressions must be specified formally via axioms, for example in the case of '**part_of**' by axioms of mereology (the theory of part and whole: see below), and in the case of '**earlier**' by axioms governing a linear order [17]. The relation **located_in **will satisfy axioms to the effect that for every continuant there is some region in which it is located; **instance_of **will satisfy axioms to the effect that all classes have (at some stage in their existence) instances, and that all instances are instances of some class.

The formal machinery for reasoning with such axioms is in place, and a comprehensive set of axioms is being compiled. For the typical human user of biological ontologies, however, the listed primitive relations and associated axioms are designed to work invisibly behind the scenes. That is, they serve as part of the background framework that guides the construction and maintenance of such ontologies.

## Results

### Methodology

We employed a multi-stage methodology for the selection of the relations to be included in this ontology and for the formulation of corresponding definitions. First, a sample of researchers involved in ontology construction in the life sciences, representing different groups and including the co-authors of this paper, was asked to prepare lists of principal relations in light of their own specific experience but focusing on relations which would be: 'ontological' in the sense introduced above; 'general-purpose' in the sense that they apply across all biological domains; and also such as to manifest a high degree of universality (in the sense explained in the section 'Types of relational assertions' below). The submitted lists manifested a significant degree of overlap, which allowed us to prepare a core list in whose terms a large number of the remaining relations on the list could be simply defined.

A further constraint on the process was the goal of providing a simple formal definition for each included <class-class> relation. Those relations for which an appropriate simple definition could not be agreed upon were not included in this interim list. This includes most conspicuously relations involving analogs of the GO notion of molecular function. The relation *has_agent *was, however, included in light of a common understanding that the notion of agency would be involved in whatever candidate definition of function in biology is eventually accepted for use in OBO. This further constraint was chosen in light of the fact that our capacity to provide simple formal definitions - definitions which will at one and the same time be intelligible to ontology authors and curators and also able to support logic-based tools for automatic reasoning and consistency-checking - is the primary rationale for the methodology here advanced.

The two relations *is_a *and *part_of *were unproblematic candidates for inclusion in the resulting list (though providing simple definitions even for these relations was not, as we shall see, a simple matter). *Is_a *and *part_of *have established themselves as foundational to current ontologies. They have a central role in almost all domain ontologies, including the Foundational Model of Anatomy (FMA) [19,20], GO and other ontologies in OBO, as well as in influential top-level ontologies such as DOLCE [21] and in digitalized lexical resources such as WordNet [22].

In preparing our sample lists we drew on representatives not only of the OBO consortium but also of GALEN and the FMA (itself a candidate for inclusion in OBO). Our temporal relations draw on existing OBO practice (where *transformation_of *is a generalization of the *develops_from *relation used in OBO's cell and anatomy ontologies) and our participation relations draw on current work addressing the need to provide relations that link entities in different ontologies (for example entities in GO's process, function and component ontologies) and on an evolving Physiology Reference Ontology that is being developed in conjunction with the FMA [23], from which our spatial relations were extracted.

### The OBO Relation Ontology

The first proposed version of the OBO Relation Ontology is shown in Table 1. We shall deal here with each of the ten relations listed in Table 1 in turn, providing rigorous yet easily understandable definitions.

#### *Is_a*

It is commonly assumed in the literature of knowledge representation that the relation *is_a *(meaning 'is a subtype of') can be identified with the subset or set inclusion relation with which we are familiar from mathematical set theory [17]. **Instance_of **functions on this reading as a counterpart of the usual set-theoretic membership relation, yielding a definition of *A is_a B *along the lines of: for all *x*, if *x ***instance_of ***A*, then *x ***instance_of ***B*. Unfortunately, this reading provides at best a necessary condition for the truth of *A is_a B*. It falls short of providing a sufficient condition for two reasons. The first is because it admits cases of contingent inclusion such as: *bacterium in 90 mm × 18 mm glass Petri dish is_a bacterium*, and the second is because it fails to take account of time, so that when applied to classes of continuants it yields false positives such as *adult is_a child *(because every instance of *adult *was at some time an instance of *child*).

We resolve the first problem by admitting as *is_a *links only assertions that reflect truths of biological science - assertions involving genuine biological class names (such as 'enzyme' or 'apoptosis') rather than, for example, commercial or indexical names (such as 'bacterium in this Petri dish'). The second problem we resolve by exploiting our machinery for taking account of time in the assertion of *is_a *relations involving continuants.

We can then define:

*C is_a C*_1 _= [definition] for all *c*, *t*, if *c ***instance_of ***C ***at ***t *then *c ***instance_of ***C*_1 _**at ***t*.

*P is_a P*_1 _= [definition] for all *p*, if *p ***instance_of ***P *then *p ***instance_of ***P*_1_.

Note how the device of logical quantifiers (for all ..., for some ...) allows us to refer to instances 'in general' - which means without the need to call on the proper names or indexical expressions (such as 'this' or 'here') which we use when referring to instances 'in specific'. Note also how instantiation for continuants involves a temporal argument. This reflects the fact that continuants, but not processes, can instantiate different classes in the course of their existence and yet preserve their identity.

For simplicity of expression we shall henceforth write '*Cct*' and '*Pp*', as abbreviations for: '*c ***instance_of *C *at *t ***' and '*p ***instance_of ***P *', respectively.

#### *Part_of*

**Parthood as a relation between instances.** The primitive instance-level relation *p ***part_of ***p*_1 _is illustrated in assertions such as: this instance of *rhodopsin mediated phototransduction ***part_of **this instance of *visual perception*.

This relation satisfies at least the following standard axioms of mereology: reflexivity (for all *p*, *p ***part_of ***p*); anti-symmetry (for all *p*, *p*_1_, if *p ***part_of ***p*_1 _and *p*_1 _**part_of ***p *then *p *and *p*_1 _are identical); and transitivity (for all *p*, *p*_1_, *p*_2_, if *p ***part_of ***p*_1 _and *p*_1 _**part_of ***p*_2_, then *p ***part_of ***p*_2_). Analogous axioms hold also for parthood as a relation between spatial regions.

For parthood as a relation between continuants, these axioms need to be modified to take account of the incorporation of a temporal argument. Thus for example the axiom of transitivity for continuants will assert that if *c ***part_of ***c*_1 _**at ***t *and *c*_1 _**part_of ***c*_2 _**at ***t*, then also *c ***part_of ***c*_2 _at *t*.

**Parthood as a relation between classes.** To define *part_of *as a relation between classes we again need to distinguish the two cases of continuants and processes, even though the explicit reference to instants of time now falls away. For continuants, we have *C part_of C*_1 _if and only if any instance of *C *at any time is an instance-level part of some instance of *C*_1 _at that time, as for example in: *cell nucleus part_ of cell*.

Formally:

*C part_of C*_1 _= [definition] for all *c*, *t*, if *Cct *then there is some *c*_1_ such that *C*_1_*c*_1_*t *and *c ***part_of ***c*_1 _**at ***t*.

Note the 'all-some' structure of this definition, a structure which will recur in almost all the relations treated here.

*C part_of C*_1 _defines a relational property of permanent parthood for *C*s. It tells us that *C*s, whenever they exist, exist as parts of *C*_1_s. We can also define in the obvious way *C temporary_part_of C*_1 _(every *C *exists at some time in its existence as part of some *C*_1_) and also *C initial_part_of C*_1 _(every *C *is such that it begins to exist as part of some instance of *C*_1_).

For processes, we have by analogy, *P part_of P*_1 _if and only if any instance of *P *is an instance-level part of some instance of *P*_1_, as for example in: *M phase part_of cell cycle *or *neuroblast cell fate determination part_of neurogenesis*. Formally:

*P part_of P*_1 _= [definition] for all *p*, if *Pp *then there is some *p*_1 _such that: *P*_1_*p*_1 _and *p ***part_of ***p*_1_.

An assertion to the effect that *P part_of P*_1 _thus tells us that *P*s in general are in every case such as to exist as parts of *P*_1_s. *P*_1_s themselves, however, may exist without having *P*s as parts (consider: *menopause part_of aging*).

Note that *part_of *is in fact two relations, one linking classes of continuants, the other linking classes of processes. While both of the mentioned relations are transitive, this does not mean that *part_of *relations could be inferred which would cross the continuant-process divide.

#### *Located_in*

**Location as a relation between instances.** The primitive instance-level relation *c ***located_in ***r ***at ***t *reflects the fact that each continuant is at any given time associated with exactly one spatial region, namely its exact location [24]. Following [25] we can use this relation to define a further instance-level location relation - not between a continuant and the region which it exactly occupies, but rather between one continuant and another. *c *is located in *c*_1_, in this sense, whenever the spatial region occupied by *c *is **part_of **the spatial region occupied by *c*_1_. Formally:

*c ***located_in ***c*_1 _at *t *= [definition] for some *r*, *r*_1_, *c ***located_in ***r ***at ***t *and *c*_1 _**located_in ***r*_1 _**at ***t *and *r ***part_of ***r*_1_.

Note that this relation comprehends both the relation of exact location between one continuant and another which obtains when *r *and *r*_1 _are identical (for example, when a portion of fluid exactly fills a cavity), as well as those sorts of inexact location relations which obtain, for example, between brain and head or between ovum and uterus.

**Location as a relation between classes.** To define location as a relation between classes - represented by sentences such as *ribosome located_in cytoplasm*, *intracellular located_in cell *- we now set:

*C located_in C*_1 _= [definition] for all *c*, *t*, if *Cct *then there is some *c*_1 _such that *C*_1_*c*_1_*t *and *c ***located_in ***c*_1 _**at ***t*.

Note that *C located_in C*_1 _is an assertion about *C*s in general, which does not tell us anything about *C*_1_s in general (for example, that they have *C*s located in them).

#### *Contained_in*

If *c ***part_of ***c*_1 _**at ***t *then we have also, by our definition and by the axioms of mereology applied to spatial regions, *c ***located_in ***c*_1 _**at ***t*. Thus, many examples of instance-level location relations for continuants are in fact cases of instance-level parthood. For material continuants location and parthood coincide. Containment is location not involving parthood, and arises only where some immaterial continuant is involved. To understand this relation, we first define overlap for continuants as follows:

*C*_1 _**overlap ***c*_2 _**at ***t *= [definition] for some *c*, *c ***part_of ***c*_1 _**at ***t *and *c ***part_of ***c*_2 _**at ***t*.

The containment relation on the instance level can then be defined as follows:

*c ***contained_in ***c*_1 _**at ***t *= [definition] *c ***located_in ***c*_1 _**at ***t *and not *c ***overlap ***c*_1 _**at ***t*.

On the class level this yields:

*C contained_in C*_1 _= [definition] for all *c*, *t*, if *Cct *then there is some *c*_1 _such that: *C*_1_*c*_1_*t *and *c ***contained_in ***c*_1 _**at ***t*.

Containment obtains in each case between material and immaterial continuants, for instance: *lung contained_in thoracic cavity*; *bladder contained_in pelvic cavity*. Hence containment is not a transitive relation.

#### *Adjacent_to*

**Figure 1:**
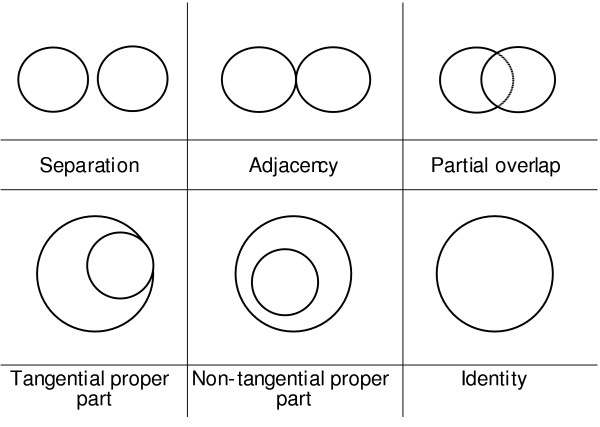
Standard mereotopological relations between spatial regions.

We can define additional spatial relations by appealing to the primitive **adjacent_to**, a relation of proximity between disjoint continuants. **Adjacent_to **satisfies some of the axioms governing the relation referred to in the literature of qualitative topology as 'external connectedness' [26]. Analogs of other mereotopological relations (qualitative relations between spatial regions involving parthood, boundary and connectedness) (Figure 1) can also be defined, and these too can be applied to the material and immaterial continuants which occupy such regions on the instance level.

We define overlap for spatial regions as follows:

*r*_1 _**overlap ***r*_2 _= [definition] for some *r*, *r ***part_of ***r*_1 _and *r ***part_of ***r*_2_.

We then assert axiomatically that *r*_1 _**adjacent_to ***r*_2 _implies not *r*_1 _**overlap ***r*_2_

We can then define the counterpart relation of adjacency between classes as follows:

*C adjacent_to C*_1 _= [definition] for all *c*, *t*, if *Cct*, there is some *c*_1 _such that: *C*_1_*c*_1_*t *and *c ***adjacent_to ***c*_1 _**at ***t*.

Note that *adjacent_to *as thus defined is not a symmetric relation, in contrast to its instance-level counterpart. For it can be the case that *C*s are in general such as to be adjacent to instances of *C*_1 _while no analogous statement holds for *C*_1_s in general in relation to instances of *C*. Examples are:

nuclear membrane adjacent_to cytoplasm

seminal vesicle adjacent_to urinary bladder

*ovary adjacent_to parietal pelvic peritoneum*.

We can, however, very simply define a symmetric relation of co-adjacency on the class level as follows:

*C*_1 _*co-adjacent_to C*_2 _= [definition] *C*_1 _*adjacent_to C*_2 _and *C*_2 _*adjacent_to C*_1_.

Examples are:

inner layer of plasma membrane co-adjacent_to outer layer of plasma membrane

right pulmonary artery co-adjacent_to right principal bronchus

*urinary bladder of female co-adjacent_to parietal peritoneum of female pelvis*.

#### *Transformation_of*

**Figure 2:**
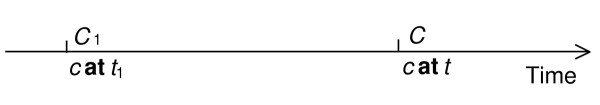
Transformation.

When an embryonic oenocyte (a type of insect cell) is transformed into a larval oenocyte, one and the same continuant entity preserves its identity while instantiating distinct classes at distinct times. The class-level relation *transformation_of *obtains between continuant classes *C *and *C*_1 _wherever each instance of the class *C *is such as to have existed at some earlier time as an instance of the distinct class *C*_1 _(see Figure 2). This relation is illustrated first of all at the molecular level of granularity by the relation between *mature RNA *and the *pre-RNA *from which it is processed, or between (*UV-induced*) *thymine-dimer *and *thymine dinucleotide*. At coarser levels of granularity it is illustrated by the transformations involved in the creation of red blood cells, for example, from *reticulocyte *to *erythrocyte*, and by processes of development, for example, from *larva *to *pupa*, or from (post-gastrular) *embryo *to *fetus *[27] or from *child *to *adult*. It is also manifest in pathological transformations, for example, of *normal colon *into *carcinomatous colon*. In each such case, one and the same continuant entity instantiates distinct classes at different times in virtue of phenotypic changes.

As definition for this relation we offer:

*C transformation_of C*_1 _= [definition] *C *and *C*_1 _for all *c*, *t*, if *Cct*, then there is some *t*_1 _such that *C*_1_*ct*_1_, and *t*_1 _**earlier ***t*, and there is no *t*_2 _such that *Cct*_2 _and *C*_1_*ct*_2_.

That is to say, the class *C *is a transformation of the class *C*_1 _if and only if every instance *c *of *C *is at some earlier time an instance of *C*_1_, and there is no time at which it is an instance of both *C *and *C*_1_. (The final clause, which asserts that *C *and *C*_1 _do not share instances at a time, is inserted in order to rule out, for example, *adult human transformation_of human*.)

Note that *C transformation_of C*_1 _is a statement about *C*s in general. It does not tell us of *C*_1_s in general that each gives rise to some *C *which stands to it in a *transformation_of *relation.

#### *Derives_from*

**Derivation as a relation between instances.** The temporal relation of derivation is more complex. Transformation, on the instance level, is just the relation of identity: each adult is identical to some child existing at some earlier time. Derivation on the instance-level is a relation holding between non-identicals. More precisely, it holds between distinct material continuants when one succeeds the other across a temporal divide in such a way that at least a biologically significant portion of the matter of the earlier continuant is inherited by the later. Thus we will have axioms to the effect that from *c ***derives_from ***c*_1 _we can infer that *c *and *c*_1 _are not identical and that there is some instant of time *t *such that *c*_1 _exists only prior to and *c *only subsequent to *t*. We will also be able to infer that the spatial region occupied by *c *as it begins to exist at *t *overlaps with the spatial region occupied by *c*_1 _as it ceases to exist in the same instant.

**Figure 3:**
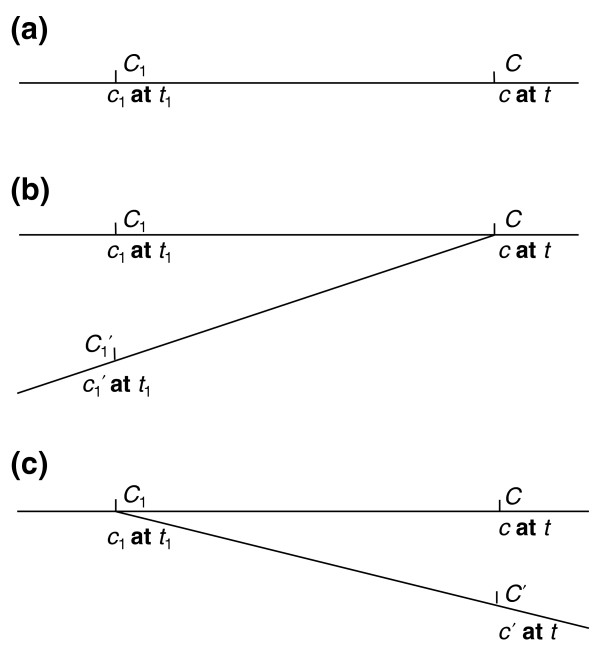
Three simple cases of derivation. **(a) **Continuation; **(b) **fusion; **(c) **fission.

Three simple kinds of instance-level derivation can then be distinguished (Figure 3): first, the succession of one single continuant by another single continuant across a temporal threshold (for example, this blastocyst derives from this zygote); second, the fusion of two or more continuants into one continuant (for example, this zygote derives from this sperm and from this ovum); and third, the fission of an earlier single continuant to create a plurality of later continuants (for example, these promyelocytes derive from this myeoloblast). In all cases we have two continuants *c *and *c*_1 _which are such that *c *begins to exist at the same instant of time at which *c*_1 _ceases to exist, and at least a significant portion of the matter of *c*_1 _is inherited by its successor *c*.

Derivation of the first type is still essentially weaker than transformation, for the latter involves the identity of the continuant instances existing on either side of the relevant temporal divide. In derivation of the second type, the successor continuant takes the bulk of its matter from a plurality of precursors, where in cases of the third type, the bulk of the matter of a single precursor continuant is shared among a plurality of successors. We can also represent more complex cases where transformation and an analog of derivation are combined, for example in the case of *budding *in yeast [27], where one continuant continues to exist identically through a process wherein a second continuant floats free from its host; or in *absorption*, where one continuant continues to exist identically through a process wherein it absorbs another continuant, for example through digestion.

**Derivation as a relation between classes.** To avoid troubling counter-examples, the relation of derivation we are seeking on the class level must be defined in two steps. First, the class-level counterpart of the relation of derivation on the instance level is identified as a relation of immediate derivation:

*C derives_immediately_from C*_1 _= [definition] for all *c*, *t*, if *Cct*, then there is some *c*_1_,*t*_1_, such that: *t*_1 _**earlier ***t *and *C*_1_*c*_1_*t*_1 _and *c ***derives_from ***c*_1_.

The more general class level derivation relation must then be defined in terms of chains of immediate derivation relations, as follows:

*C derives_from C*_1 _= [definition] there is some sequence *C *= *C*_*k*_, *C*_*k*-1_, ..., *C*_2_, *C*_1_, such that for each *C*_*i *_(1 ≤ i < k), *C*_*i*+1 _*derives_immediately_from C*_*i*_.

In this way we can represent cases of derivation involved in the formation of lineages where there occurs a sequence of cell divisions or speciation events.

#### *Preceded_by*

With the primitive relations **has_participant **and **earlier **at our disposal we can define the instance-level relation *p ***occurring_at ***t *as follows:

*p ***occurring_at ***t *= [definition] for some *c*, *p ***has_participant ***c ***at ***t*.

We can then define:

*c ***exists_at ***t *= [definition] for some *p, p ***has_participant ***c ***at ***t*

*p ***preceded_by ***p*_1 _= [definition] for all *t, t*_1_, if *p ***occurring_at ***t *and *p*_1 _**occurring_at ***t*_1_, then *t*_1 _**earlier ***t*

*t ***first_instant ***p *= [definition] *p ***occurring_at ***t *and for all *t*_1_, if *t*_1 _**earlier ***t*, then not *p ***occurring_at ***t*_1_

*t ***last_instant ***p *= [definition] *p ***occurring_at ***t *and for all *t*_1_, if *t ***earlier ***t*_1_, then not *p *occurring_at *t*_1_

*p ***immediately_preceded_by ***p*_1 _= [definition] for some *t, t ***first_instant ***p *and *t ***last_instant ***p*_1_.

At the class level we have:

*P preceded_by P*_1 _= [definition] for all *p*, if *Pp *then there is some *p*_1 _such that *P*_1_*p*_1_and *p ***preceded_by ***p*_1_.

An example is: *translation preceded_by transcription*; *aging preceded_by development *(not however *death preceded_by aging*). Where *derives_from *links classes of continuants, *preceded_by *links classes of processes. Clearly, however, these two relations are not independent of each other. Thus if cells of type *C*_1 _*derive_from *cells of type *C*, then any cell division involving an instance of *C*_1 _in a given lineage is *preceded_by *cellular processes involving an instance of *C*.

The assertion *P preceded_by P*_1 _tells us something about *P*s in general: that is, it tells us something about what happened earlier, given what we know about what happened later. Thus it does not provide information pointing in the opposite direction, concerning instances of *P*_1 _in general; that is, that each is such as to be succeeded by some instance of *P*. Note that an assertion to the effect that *P preceded_by P*_1 _is rather weak; it tells us little about the relations between the underlying instances in virtue of which the *preceded_by *relation obtains. Typically we will be interested in stronger relations, for example in the relation *immediately_preceded_by*, or in relations which combine *preceded_by *with a condition to the effect that the corresponding instances of *P *and *P*_1 _share participants, or that their participants are connected by relations of derivation, or (as a first step along the road to a treatment of causality) that the one process in some way affects (for example, initiates or regulates) the other.

#### *Has_participant*

**Has_participant **is a primitive instance-level relation between a process, a continuant, and a time at which the continuant participates in some way in the process. The relation obtains, for example, when this particular process of oxygen exchange across this particular alveolar membrane **has_participant **this particular sample of hemoglobin at this particular time.

To define the class-level counterpart of the participation relation we set:

*P has_participant C *= [definition] for all *p*, if *Pp *then there is some *c*, *t *such that *Cct *and *p ***has_participant ***c ***at ***t*.

Examples are:

cell transport has_participant cell

death has_participant organism

*breathing has_participant thorax*.

Once again, *P has_participant C *provides information only about *P*s in general (that is, that they require instances of *C *as bearers).

#### *Has_agent*

Special types of participation can be distinguished according to whether a continuant is agent or patient in a process (for a survey see [28].) Here we focus on the factor of agency, which is involved, for example, when an adult engages in adult walking behavior. It is not involved when the same adult is the victim of an infection. Synonyms of 'is agent in' include: 'actively participates in', 'does', 'executes', 'performs', and so forth.

We introduce the primitive instance-level relation **has_agent**, which obtains between a process, a continuant and a time whenever the continuant is a participant in the process and is at the same time directly causally responsible for its occurrence. Thus we have an axiom to the effect that agency implies participation: for all *p*, *c*, *t*, if *p ***has_agent ***c ***at ***t*, then *p ***has_participant ***c ***at ***t*. In addition we will have axioms to the effect that only material continuants can fill the agent role, that if *c *fills the agent role at *t*, then *c *must have existed at times earlier than *t*, that it must exercise its agent role for an interval of time including *t*, and so on.

We can then define the class-level relation *has_agent *by stipulating:

*P has_agent C *= [definition] for all *p*, if *Pp *then there is some *c*, *t *such that *Cct *and *p ***has_agent ***c ***at ***t*

This relation gives us the means to capture the directionality (the from-to) nature of biological processes such as signaling, transcription, and expression, via assertions, for example, to the effect that in an interaction between molecules of types *m*_1 _and *m*_2 _it is molecules of the first type that play the role of agent.

One privileged type of agency consists in the realization of a biological function. To say that a continuant has a function is to assert, in first approximation, that it is predisposed (has the potential, the casual power) to cause (to realize as agent) a process of a certain type. Thus to say that your heart has the function: *to pump blood *is to assert that your heart is predisposed to realize as agent a process of the type *pumping blood *[29]. Regulation, promotion, inhibition, suppression, activation, and so forth, are among the varieties of agency that fall under this heading.

On the other hand, many processes - such as metabolic reactions involving enzymes, cofactors, and metabolites - involve no clear factor of agent participation, but rather require more nuanced classifications of the roles of participants - as acceptors or donors, for example. Hence the *has_agent *relation should be used in curation with special care. It should be borne in mind in this connection that agency is in every case a matter of the imposition of direct causal influence of a continuant in a process (a constraint that is designed to rule out inheritance of agency along causal chains), and also that (by our definition) only continuants can be agents. Where biologists describe processes as agents, for example, in talking about the effects of diffusion in development and differentiation, such phenomena are of a type that call for an expansion of our proposed Relation Ontology in the direction, again, of a treatment of the factor of causality.

## Discussion

### The logic of biological relations

#### Inverse and reciprocal relations

The inverse of a relation R is defined as that relation which obtains between each pair of relata of R when taken in reverse order. Inverses can be unproblematically defined for all instance-level relations. What, then, of inverses for class-level relations? The inverse relation for *is_a *can be defined trivially as follows:

*A has_subclass B *= [definition] *B is_a A*.

For the remaining class-level relations on our list, in contrast, the issue of corresponding inverses is more problematic [7]. Thus, while we have the true relational assertion *human testis part_of human *- which means that all instances of *human testis *are part of instances of some *human *- there is no corresponding true relational assertion linking instances of *human *to instances of *human testis *as their parts. For these remaining relations we need to work not with inverses but rather with what, following GALEN, we can call reciprocal relations. These are defined using the same family of instance-level primitives we introduced earlier. As reciprocal relations for the two varieties of *part_of *we have:

*C has_part C*_1 _= [definition] for all *c*, *t*, if *Cct *then there is some *c*_1 _such that *C*_1_*c*_1_*t *and *c*_1 _**part_of ***c ***at ***t*

*P has_part P*_1 _= [definition] for all *p*, if *Pp *then there is some *p*_1 _such that *P*_1_*p*_1 _and *p*_1 _**part_of ***p*

Note that from *A part_of B *we cannot infer that *B has_ part A*; similarly, from *A has_ part B *we cannot infer that *B part_of A*. Thus *cell nucleus part_of cell*, but not *cell has_part cell nucleus*; *running has_ part breathing*, but not *breathing part_of running*. A third significant relation conjoining *part_of *and *has_part *can be defined as [6,30]:

*C integral_part_of C*_1 _= [definition] *C part_of *C_1 _and *C*_1 _*has_part C*.

For *contained_in *we have similarly the reciprocal relation:

*C contains C*_1 _= [definition] for all *C*, *t*, if *Cct *then there is some *c*_1 _such that: *C*_1_*c*_1_*t *and *c ***located_in ***c ***at ***t*

For participation we can usefully define two alternative reciprocal relations:

*C sometimes_ participates_in P *= [definition] for all *c *there is some *t *and some *p *such that *Cct *and *Pp *and *p ***has_participant ***c ***at ***t*

*C always_participates_in P *= [definition] for all *c*, *t*, if *Cct *then there is some *p *such that *Pp *and *p ***has_participant ***c ***at ***t*

We can also define, for example, what it is for continuants of a given type to participate at every stage in a process of a given type. Thus if a sperm participates in the penetration of an ovum, then it does so throughout the penetration.

### Types of relational assertions

In light of the above, we can now observe certain differences in what we might call the relative universality of class-level relational assertions. There are many cases, above all involving *is_a *relations, where relational assertions hold with a maximal degree of universality, which means that they hold for every instance of the classes in question because they are a matter of analytic connections, that is, connections resting on the compositional nature of the class terms involved [10], as, for example, in: *eukaryotic cell is_a cell*, or *adult walking behavior has_participant adult*. (Contrast, *adult participates_in adult walking behavior*.)

There are also other kinds of statements enjoying a high degree of universality, for example: *penetration of ovum has_participant sperm*. The first of our two corresponding reciprocal statements - *sperm participates_in penetration of ovum *- is in contrast true only in relation to certain isolated instances of *sperm*, and the second of our reciprocal statements - *sperm always_participates_in penetration of ovum *- is true in relation to no instances at all.

It then seems reasonable to insist that biomedical ontologies should reflect those sorts of biological assertions that enjoy a high degree of universality (typically assertions involving just one of each pair of reciprocal relations).

### Tools for ontology curation

We hope that, by providing clear and unambiguous specifications of what the class-level relational expressions used in biological ontologies mean, our formal definitions will assist curators engaged in ontology creation and maintenance. The corresponding definitions are summarized in Table 2, which also contains representative examples for each of the relations distinguished.

Our definitions are designed to ensure that the corresponding general-purpose relational expressions are used in a uniform way in all biological ontologies. In this way we shall be in a position to contribute to the realization of the goal of bringing about a high degree of interoperability even where ontologies are produced by different groups and for different purposes. These definitions are designed also to enable the automatic detection of errors in biomedical ontologies, for example by allowing the construction of extensions of OBO-Edit and similar tools with the facility to test whether given relations are employed in an ontology in such a way as to involve relata of the appropriate types [31] or in such a way as to have the formal characteristics, such as transitivity or reflexivity, dictated by the definitions (Table 3). The framework can also support reasoning applications designed to enable the automated derivation of information from existing bodies of knowledge - for example to infer the parts of a given cell continuant via the traversal of a *part_of *hierarchy - including instance-based knowledge derived from the clinical record.

## Conclusion

The Relation Ontology outlined above arose through collaboration between formal ontologists and biologists in the OBO, FMA and GALEN research groups and also incorporates suggestions from a number of other authors and curators of biomedical ontologies. It is designed to be large enough to overcome some of the problems arising in GO and similar systems as a result of the paucity of resources available hitherto for expressing relations between the classes in such ontologies [32]. It is this paucity of resources, above all, which gives rise to cases of multiple inheritance in GO as presently constructed, and we note here that multiple inheritance often goes hand in hand with errors in ontology construction not least because it encourages a relaxed reading of *is_a *(often a reading which involves the assertion of *is_a *relations which erroneously cross the divide between different ontological categories) [5,33]. Our present framework can contribute to error resolution not only by dictating a common interpretation of *is_a *which can serve as orientation for ontology authors and curators in their future work, but also by providing richer resources for the assertion of class-class relations within and between ontologies in such a way that the appeal to contrived and error-prone *is_a *relations can be more easily avoided.

At the same time our suite of relations has been designed to be sufficiently small to attract wide acceptance in a range of different types of life-science communities. Where the latter use further, general-purpose or domain-specific relations of their own, we plan in due course to subject such relations to the same kind of analysis as presented here in order to preserve interoperability. The Relation Ontology has been incorporated into the OBO ontology library [34] and curators of the GO and FMA ontologies and also of the ChEBI chemical entities vocabulary [35] are already applying the relevant parts of the ontology in their work. The ontology has already been used to find errors not only in GO but also in SNOMED [36]. It is also being applied systematically in evaluations of the NCI Thesaurus [37] and the UMLS (Unified Medical Language System) Semantic Network of the National Library of Medicine. We are currently testing methodologies to obtain reliable quantitative evaluations of the utility of the proposed framework for purposes of ontology authoring and also for use in annotation and reasoning. We are also testing ways in which the framework can be expanded through the admission of pre-coordinated disjunctions (for example: *either derivation or transformation*), which can allow the coding of information in those cases where the precise nature of the relations involved is insufficiently clear to allow unique assignment.

The Relation Ontology will be evaluated on two levels. First, on whether it succeeds in preventing those characteristic kinds of errors which have been associated with a poor treatment of relations in biomedical ontologies in the past. Second, and more important, on whether it helps to achieve greater interoperability of biomedical ontologies and thus to improve reasoning about biological phenomena.

## Figures and Tables

**Table 1:** First version of the OBO Relation Ontology

Foundational relations
*is_a*
*part_of*

Spatial relations (connecting one entity to another in terms of relations between the spatial regions they occupy)
*located_in*
*contained_in*
*adjacent_to*

Temporal relations (connecting entities existing at different times)
*transformation_of*
*derives_from*
*preceded_by*

Participation relations (connecting processes to their bearers)
*has_participant*
*has_agent*

**Table 2:** Definitions and examples of class-level relations

Relations and relata	Definitions	Examples
*C is_a C*_1_; *C*s and *C*_1_s are continuants	Every *C *at any time is at the same time a *C*_1_	*myelin is_a lipoprotein*
		*serotonin is_a biogenic amine*
		*mitochondrion is_a membranous cytoplasmic organelle*
		*protein kinase is_a kinase*
		*DNA is_a nucleic acid*
		
*P is_a P*_1_; *P*s and *P*_1_s are processes	Every *P *is a *P*_1_	*endomitosos is_a DNA replication*
		*catabolic process is_a metabolic process*
		*photosynthesis is_a physiological process*
		*gonad development is_a organogenesis*
		*intracellular signaling cascade is_a signal transduction*
		
*C part_of C*_1_; *C*s and *C*_1_s are continuants	Every *C *at any time is part of some *C*_1 _at the same time	*mitochondrial matrix part_of mitochondrion*
		*microtubule part_of cytoskeleton*
		*nuclear pore complex part_of nuclear membrane*
		*nucleoplasm part_of nucleus*
		*promotor part_of gene*
		
*P part_of P*_1_; *P*s and *P*_1_s are processes	Every *P *is part of some *P*_1_	*gastrulation part_of embryonic development*
		*cystoblast cell division part_of germ cell development*
		*cytokinesis part_of cell proliferation*
		*transcription part_of gene expression*
		*neurotransmitter release part_of synaptic transmission*
		
*C located_in C*_1_; *C*s and *C*_1_s are continuants	Every *C *at any given time occupies a spatial region which is part of the region occupied by some *C*_1 _at the same time	*66s pre-ribosome located_in nucleolus*
		*intron located_in gene*
		*nucleolus located_in nucleus*
		*membrane receptor located_in cell membrane*
		*chlorophyll located_in thylakoid*
		
*C contained_in C*_1_; *C*s are material continuants, *C*_1_s are immaterial continuants (holes, cavities)	Every *C *at any given time is located in but shares no parts in common with some *C*_1 _at the same time	*thoracic aorta contained_in posterior mediastinal cavity*
		*cytosol contained_in cell compartment space*
		*thylakoid contained_in chloroplast membrane*
		*synaptic vesicle contained_in neuron*
		
*C adjacent_to C*_1_; *C*s and *C*_1_s are continuants	Every *C *at any time is proximate to some *C*_1 _at the same time	*Golgi apparatus adjacent_to endoplasmic reticulum*
		*intron adjacent_to exon*
		*cell wall adjacent_to cytoplasm*
		*periplasm adjacent_to plasma membrane*
		*presynaptic membrane adjacent_to synaptic cleft*
*C transformation_of C*_1_; *C*s and *C*_1_s are material continuants	Every *C *at any time is identical with some *C*_1 _at some earlier time	*facultative heterochromatin transformation_of euchromatin*
		*mature mRNA transformation_of pre-mRNA*
		*hemosiderin transformation_of hemoglobin*
		*red blood cell transformation_of reticulocyte*
		*fetus transformation_of embryo*
		
*C derives_from C*_1_; *C*s and *C*_1_s are material continuants	Every *C *is such that in the first moment of its existence it occupies a spatial region which overlaps the spatial region occupied by some *C*_1 _in the last moment of its existence	*plasma cell derives_from B lymphocyte*
		*fatty acid derives_from triglyceride*
		*triple oxygen molecule derives_from oxygen molecule*
		*Barr body derives_from X-chromosome*
		*mammal derives_from gamete*
		
*P preceded_by P*_1_; *P*s and *P*_1_s are processes	Every *P *is such that there is some earlier *P*_1_	*translation preceded_by transcription*
		*meiosis preceded_by chromosome duplication*
		*cytokinesis preceded_by DNA replication*
		*apoptotic cell death preceded_by nuclear chromatin degradation*
		*digestion preceded_by ingestion*
		
*P has_participant C*; *P*s are processes, *C*s are continuants	Every *P *involves some *C *as participant	*mitochondrial acetylCoA formation has_participant pyruvate dehydrogenase complex*
		*translation has_participant amino acid*
		*photosynthesis has_participant chlorophyll*
		*apoptosis has_participant cell*
		*cell division has_participant chromosome*
		
*P has_agent C*; *P*s are processes, *C*s are material continuants	Every *P *involves some *C *as agent (the *C *is involved in and is causally responsible for the *P*)	*gene expression has_agent RNA polymerase*
		*signal transduction has_agent receptor*
		*pathogenesis has_agent pathogen*
		*transcription has_agent RNA polymerase*
		*translation has_agent ribosome*

**Table 3:** Some properties of the relations in the OBO Relation Ontology

Relation	Transitive	Symmetric	Reflexive	Antisymmetric
*is_a*	+	-	+	+
*part_of*	+	-	+	+
*located_in*	+	-	+	-
*contained_in*	-	-	-	-
*adjacent_to*	-	-	-	-
*transformation_of*	+	-	-	-
*derives_ from*	+	-	-	-
*preceded_by*	+	-	-	-
*has_participant*	-	-	-	-
*has_agent*	-	-	-	-
